# Dentists' Register

**Published:** 1888-11

**Authors:** 


### Dentists' Register.

At the last meeting of the American Dental Association, held at Louisville in August, a resolution was offered and passed, appointing a committee to take into consideration the feasibility of securing a complete list of the graduates of all the Dental Colleges of the United States, with the time of graduation, the length of time in practice, and the present address, especially of those residing in this country. A list of those who have been licensed by examining boards might with propriety be embraced in such a register. Such a work as this would be of very great interest to every progressive member of the profession, and would be a great aid in certain lines of investigation and study. The preparation of such a directory would involve great labor, but if the co-operation of the profession generally could be enlisted, the work would be comparatively easy. A complete directory of the medical profession of this country was prepared in 1886, and has in many ways been of incalculable benefit. Such a Register of Dentists must be available in the near future.

